# Causes of mortality across different immigrant groups in Northeastern Italy

**DOI:** 10.7717/peerj.975

**Published:** 2015-05-21

**Authors:** Ugo Fedeli, Eliana Ferroni, Mara Pigato, Francesco Avossa, Mario Saugo

**Affiliations:** Epidemiological Department, Veneto Region, Italy; Passaggio Gaudenzio, 1-35131 Padova (PD), Italy

**Keywords:** Mortality, Immigrants

## Abstract

**Background.** Despite massive immigration towards Southern Europe in the last two decades, data on mortality by cause among immigrants in Italy are scarce. The aim of this study was to investigate mortality from all and from specific causes of death among immigrants residing in the Veneto Region (Northeastern Italy).

**Methods.** Mortality records for the period 2008–2013 were extracted from the regional archive of causes of death, whereas population data were obtained from the 2011 Italian census. Immigrants were grouped by area of provenience based on the information on country of citizenship available both in mortality and census data. Standardized Mortality Ratios (SMR) with 95% Confidence Intervals (CI) were computed for the period 2008–2013 in subjects aged 20–59 years, with rates of Italian citizens as a reference.

**Results.** Overall mortality was reduced both in male (SMR 0.86, CI [0.80–0.92]) and female immigrants (SMR 0.72, CI [0.65–0.78]), although an increased risk was observed for subjects from Sub-Saharan Africa. Significantly raised SMR for circulatory diseases were found among Sub-Saharan Africans and Southern Asians in both genders. Sub-Saharan Africans experienced a higher risk of death, especially from cerebrovascular diseases: SMR 4.78 (CI [2.67–7.89]) and SMR 6.09 (CI [1.96–14.2]) in males and females, respectively. Among Southern Asians, the increase in mortality from ischemic heart diseases reached statistical significance in males (SMR 2.53, CI [1.42–4.18]). In spite of a lower risk of death for all neoplasms combined, mortality from cancer of cervix uteri was increased among immigrants (SMR 2.61, CI [1.35–4.56]), as well as for other cancer sites in selected immigrant groups. A raised mortality was found for infectious diseases in Sub-Saharan Africans (both genders), and for transport accidents among females from Eastern Europe.

**Conclusion.** Our study showed great variations in mortality by cause and area of provenience among immigrants resident in the Veneto Region and highlighted specific health issues that should be addressed through tailored efforts in chronic diseases prevention.

## Introduction

Many studies have revealed the existence of mortality differences between the native and the immigrant population, with the latter usually showing lower rates due to many factors, mainly the “healthy migrant effect,” observed both in North America and in European countries (Singh & Hiatt, 2006; Spallek et al., 2012; Boulogne et al., 2012; Scott & Timaeus, 2013; Omariba, Ng & Vissandjée, 2014; Moncho et al., 2014). In fact, immigrants represent a much healthier population in many aspects than subjects who remain in their countries of origin and those in the host country (Regidor et al., 2008). However, mortality figures are undermined by large uncertainties in population estimates (denominators of rates) for immigrants. Furthermore, in some countries higher mortality rates in immigrants were observed, particularly in specific ethnic groups and for specific causes of death, with large variations observed by age and gender (Rostila & Fritzell, 2014; Bos et al., 2007).

Literature on this issue in Italy is scarce, limited to studies investigating overall mortality or broad nosologic sectors, without distinguishing by area of provenience. In a previous report from Central Italy, mortality rates in immigrants coming from developing countries (all countries excluding North America, EU15, and a few other high-income countries) have been found to be halved with respect to the Italian population (Martini, Chellini & Sala, 2011).

We aimed at reducing this information gap, providing detailed information on mortality in the immigrant population, taking advantage of both recent Italian census data with more reliable estimates of the residents by age, gender, and country of citizenship, and of updated mortality records coded according to the International Classification of Diseases, 10th Edition (ICD-10).

## Methods

### Study design and variable definition

This descriptive study compares all-cause and cause-specific mortality between Italian and legal immigrants in the Veneto Region (Northeastern Italy). Mortality records were extracted from the regional archive of causes of death, whereas population data were obtained from the 2011 Italian Census (http://dati-censimentopopolazione.istat.it/?lang=en&SubSessionId=be388b56-a12d-4f8f-927c-367b8771bdc9).

### Selection criteria

Irregular immigrants (illegal entry or legal entry followed by overstay), as well as subjects with a short-term stay in the region for tourism, study, or work were not included due to the lack of population (denominator) data. Since the immigrant population aged ≥64 years is small, all analyses excluded elderly subjects. Furthermore, detailed comparisons between the Italian and immigrant population were restricted to residents aged 20–59 years to deal with a more homogeneous adult population.

### Setting

Italy, traditionally a country of emigration, has experienced a large amount of immigration from developing countries in the context of massive migrations toward Southern Europe in the last two decades. Immigration in Italy is mainly for the purpose of working; in particular, males from African and South Asian countries are employed mainly in industry and construction, whereas females from Eastern Europe are employed mainly in the care of elderly people. Another important reason for migration is family reunion. The contribution of asylum seekers has been increasing only in the more recent years. The Veneto Region (Northeastern Italy, about 5 million inhabitants) is a highly industrialized area, having one of the highest prevalence of foreign population in the country (10%) due to many job opportunities available before the beginning of the recent economic crisis.

### Variables definition

Both the country of birth and the country of citizenship are registered in the death certificate. From the official census, population data are available only by country of citizenship. The latter variable was therefore utilized to define the immigrant (non-Italian) population. It must be remarked that immigrants can obtain Italian citizenship by marriage or –on demand- after a minimum of 10 consecutive years of legal residence. Their children can obtain it—if born in Italy—only after their 18th birthday. Consequently in the present study the immigrant population also includes second generation immigrants (mostly limited to early pediatric age groups), and excludes subjects born abroad who acquired Italian citizenship as stated before. To deal with larger numbers, countries of citizenship were grouped by area of provenience based on macro-geographical regions and sub-regions defined by the United Nation Organization (Table 1). (http://unstats.un.org/unsd/methods/m49/m49regin.htm).

**Table 1:** Study populations. Distribution of residents in the Veneto Region by gender, age group, and citizenship: 2011 census.

	Italians	All immigrants	North Africa	Sub-Saharan Africa	South Asia	Other Asian countries	Central and South America	Eastern Europe	Other countries^a^
Males, *n*	2,144,204	220,478^*^	33,276	24,713	25,095	16,730	5,286	111,068	4,295
Males, age distribution (%)									
0–19 yrs	19%	30%	33%	30%	29%	34%	27%	30%	14%
20–59 yrs	55%	67%	64%	69%	70%	64%	71%	68%	64%
≥60 yrs	26%	2%	3%	1%	1%	2%	3%	2%	22%
Females, *n*	2,255,678	236,850^*^	27,841	17,944	17,977	17,460	10,235	138,555	6,828
Females, age distribution (%)									
0–19 yrs	17%	26%	37%	38%	35%	30%	13%	22%	8%
20–59 yrs	51%	70%	58%	61%	63%	68%	82%	74%	78%
≥60 yrs	32%	4%	4%	1%	2%	2%	5%	4%	14%

**Notes.**

* Including 15 male and 10 female stateless subjects.

a Other countries: EU15, US, Canada, Australia, New Zealand, Israel, Japan, South Korea, Iceland, Liechtenstein, Norway, San Marino, Switzerland.

### Data collection

A copy of all death certificates in the Veneto Region is routinely transmitted to the Regional Epidemiology Department for coding of the causes of death. Since 2008, all diseases mentioned in the certificate are coded in ICD-10, and the selection of the underlying cause of death is performed using the ACME (Automated Classification of Medical Entities) software, downloaded from the US National Vital Statistics System website (http://www.cdc.gov/nchs/nvss/mmds.htm). Through the 2008–2013 period, satisfactory data quality is suggested by a low percentage (6.6%) of deaths corresponding to unknown and ill-defined causes, as previously defined (Mathers et al., 2005): symptoms, signs and ill-defined conditions (ICD-10 R00–R99), injuries with intent not determined (Y10–Y34), cardiovascular diseases with indefinite diagnostic meaning (I47.2, I49.0, I46, I50, I51.4, I51.5, I51.6; I51.9, I70.9), and cancer deaths from secondary, multiple, or unspecified sites (C76, C80, C97).

### Analysis

The regional mortality archive was investigated for the period 2008–2013 (2013 provisional data covering at least 98% of regional deaths), roughly centered around census data. Examined causes of death were the main classification chapters of the ICD-10 (infectious diseases, neoplasms, circulatory diseases, external causes). The most frequent disease categories were diabetes, cardiac ischemic diseases, cerebrovascular diseases, suicide, and transport accidents. Among tumors, those with larger numbers (lung, colorectal, breast), or with a priori knowledge of a possible increased risk among immigrants (stomach, liver, cervix uteri) were investigated. To properly compare populations (foreign and native) with huge differences in age structure, standardized mortality ratios (SMR) were computed as the ratios between deaths observed in the immigrant population (as a whole and for each area of provenience), and those expected according to age- and gender-specific mortality rates registered in Italian citizens. 95% confidence intervals (CI) based on the Poisson distribution were obtained using the Byar’s approximation (Breslow & Day, 1987).

### Ethics

The analysis of causes of mortality is included among mandatory activities of the Regional Epidemiology Department according to regional law. Data used in this study were completely anonymized; thus, ethical approval was deemed unnecessary.

## Results

Table 1 shows the age structure by gender of the study populations. The age distribution of immigrants is heavily shifted towards younger age classes, being negligible the proportion of subjects aged ≥60 years, except for the small group coming from more developed countries (EU15, US and other countries). The largest immigrant group is constituted by subjects from Eastern Europe (more than 50%), but also African countries (both Northern and Sub-Saharan) and Asian countries (both Southern and other countries, mainly including China) are well represented.

**Figure 1:**
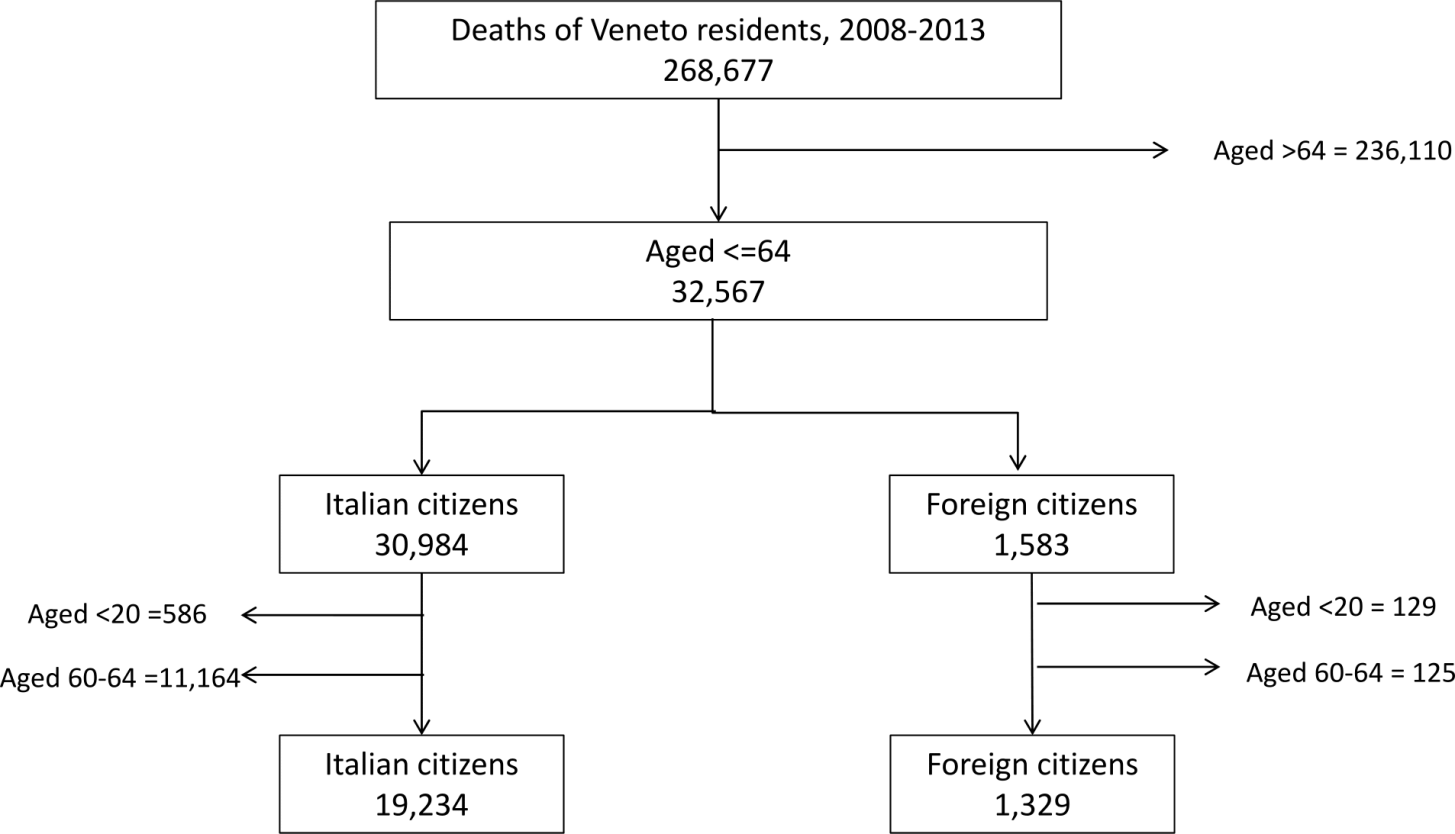
Selection of mortality records. Selection of deaths of residents in the Veneto Region by age and country of citizenship, 2008–2013.

**Figure 2:**
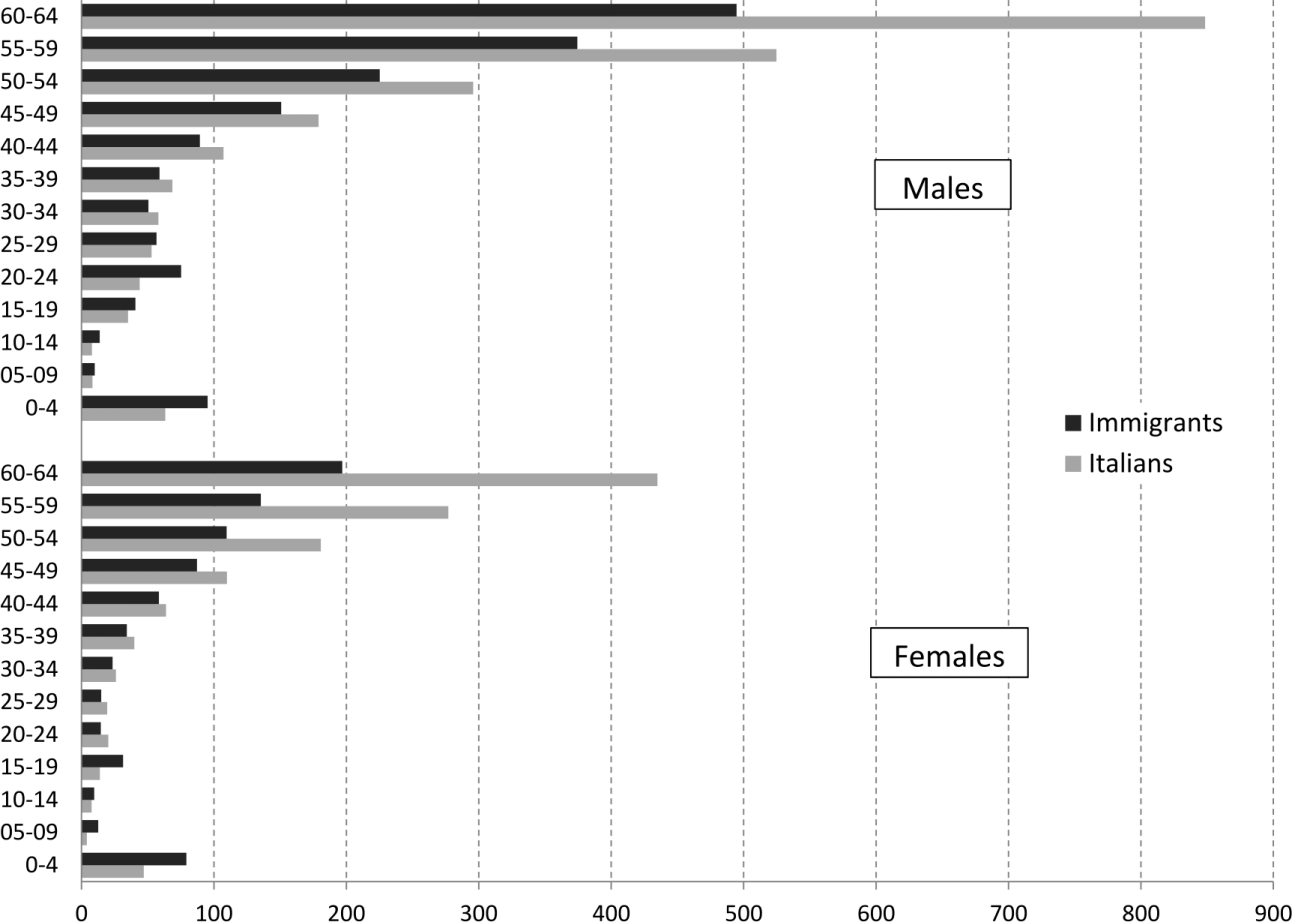
Overall mortality rates. Age-specific mortality rates (×100,000) by gender in Italian and foreign citizens. Veneto Region, 2008–2013.

In the period 2008–2013 there were 32,567 deaths in subjects aged ≤64 years, 1,583 of which occurred in immigrants (Fig. 1). In Fig. 2 it can be seen that mortality rates for all causes of death in immigrants are higher in the pediatric population in both genders. Starting from the 20–24 year (females) or the 30–34 year (males) age group, rates become higher among Italian citizens, with a widening gap with increasing age, especially among females.

In analyses restricted to the 20–59 age interval, overall mortality in immigrants compared to that observed among Italian citizens was 14% and 28% lower in males and females, respectively (Table 2). The main causes of death among male immigrants were external causes, strictly followed by neoplasms and circulatory diseases; among females, more than half of deaths were accounted for by neoplasms. With respect to the native population, cancer mortality was decreased by about 30% in immigrants of both genders (with larger decreases for colorectal and breast cancer), although a significant excess for cervical cancer could be observed. Mortality from circulatory diseases, especially cerebrovascular diseases, was increased in males, but not in females. Mortality from suicide was halved in both genders, whereas that from road accidents was similar to the Italian population in males and increased in females. Lastly, the excess risk for infectious diseases reached statistical significance among female immigrants.

**Table 2:** SMR in immigrant vs. Italian citizens. Standardized mortality ratios (SMR) with 95% Confidence Intervals (CI) in immigrants aged 20–59 years with respect to Italian citizens. Veneto Region, 2008–2013. Significantly increased SMR in bold.

Cause of death (ICD-10 codes)	Males		Females	
	*n*	SMR (CI)	*n*	SMR (CI)
All causes	863	0.86 (0.80–0.92)	466	0.72 (0.65–0.78)
Infectious diseases (A01–B99)	41	1.18 (0.85–1.60)	25	**1.64 (1.06–2.42)**
All neoplasms (C00–D48)	229	0.71 (0.62–0.80)	258	0.68 (0.60–0.76)
*Malignant neoplasm of:*				
Stomach (C16)	16	0.98 (0.56–1.59)	16	1.04 (0.60–1.70)
Colon, rectum and anus (C18–C21)	13	0.38 (0.20–0.66)	15	0.43 (0.24–0.71)
Liver and intrahepatic bile ducts (C22)	26	1.11 (0.73–1.63)	5	0.65 (0.21–1.51)
Trachea, bronchus and lung (C33–C34)	64	1.15 (0.89–1.47)	31	0.73 (0.49–1.03)
Female breast (C50)			59	0.58 (0.44–0.74)
Cervix uteri (C53 )			12	**2.61 (1.35–4.56)**
Corpus and uterus n.s. (C54–C55)			14	1.12 (0.61–1.88)
Diabetes (E10–E14)	12	0.93 (0.48–1.63)	7	1.32 (0.53–2.71)
All circulatory diseases (I00–I99)	188	1.13 (0.97–1.30)	48	0.73 (0.54–0.97)
Ischaemic heart diseases (I20–I25)	71	1.01 (0.79–1.27)	12	0.71 (0.36–1.23)
Cerebrovascular diseases (I60–I69)	36	**1.43 (1.00–1.98)**	17	0.93 (0.54–1.50)
All external causes (V01–Y89)	275	0.91 (0.80–1.02)	77	0.95 (0.75–1.19)
Transport accidents (V01–V99)	133	1.00 (0.83–1.18)	40	**1.41 (1.01–1.92)**
Suicide (X60–X84)	55	0.50 (0.38–0.66)	19	0.53 (0.32–0.83)

**Figure 3:**
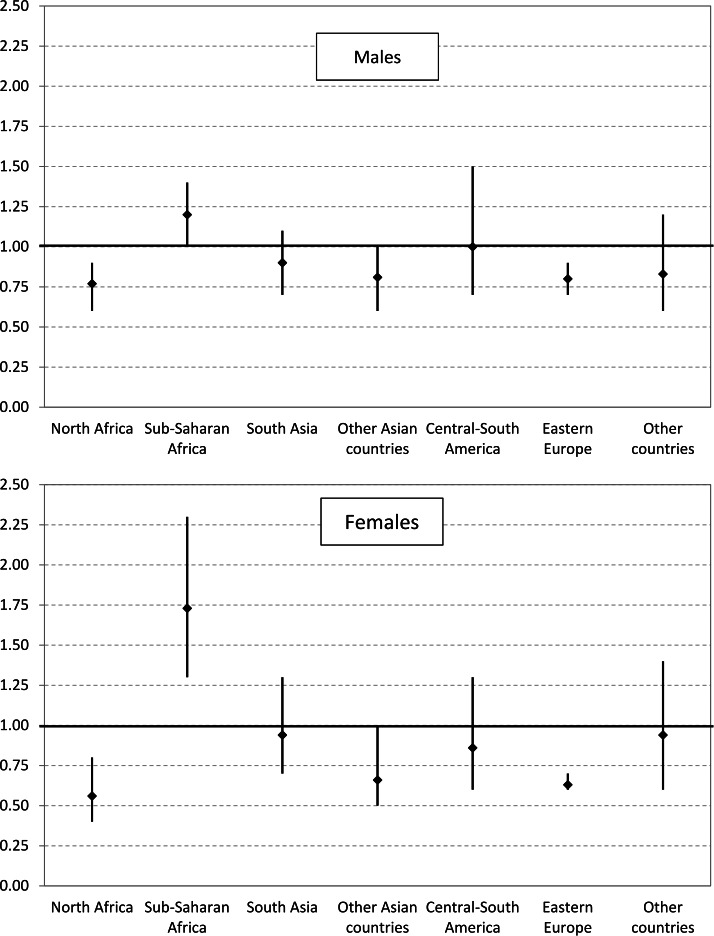
Overall mortality by area of provenience. Standardized mortality ratios (SMR) with 95% Confidence Intervals (CI) for all causes of death in immigrants aged 20–59 years with respect to Italian citizens (SMR = 1.00), by area of provenience and gender. Veneto Region, 2008–2013.

The mortality advantage observed among immigrants with respect to Italian citizens greatly varied by area of provenience (Fig. 3); in both genders, rates were significantly decreased among subjects from North Africa and Eastern Europe, while they were raised among Sub-Saharan Africans (males SMR 1.20, CI [1.01–1.41]; females SMR 1.73, CI [1.30–2.26]).

Table 3 shows variations in SMR for the leading causes of death by area of provenience (citizens from South America and from other countries are not shown due to small numbers). High risks could be recognized for circulatory diseases among immigrants from Sub-Saharan Africa (especially cerebrovascular diseases), and from South Asia (especially ischemic heart diseases), with an excess mortality observed in both genders. Notably in the largest group (Eastern Europe), mortality from circulatory diseases was decreased in females but not in males, which contributes to the gender gap in SMR estimates for circulatory disorders computed for the whole immigrant population. Overall cancer mortality was decreased among almost all immigrant groups, but specific excess risks could be detected for liver cancer (Asian countries, Sub-Saharan Africa), lung cancer (Asian countries, Eastern Europe with borderline significance), and cancer of the cervix uteri (Eastern Europe). Lastly, the increase in mortality from infectious diseases was limited to subjects from Sub-Saharan Africa, and the increase in fatal transport accidents in females was confirmed in the largest immigrant group (Eastern Europe).

**Table 3:** SMR by area of provenience. Standardized mortality ratios with 95% Confidence intervals in immigrants aged 20–59 years with respect to Italian citizens, by area of provenience and gender (M = males, F = females). Veneto Region, 2008–2013. Significantly increased SMR in bold.

		North Africa	Sub-Saharan Africa	South Asia	Asia, other	Eastern Europe
Infectious diseases	M	0.75 (0.20–1.91)	**4.35 (2.65–6.71)**	1.53 (0.49–3.57)	1.14 (0.23–3.33)	0.41 (0.16–0.85)
	F	1.68 (0.19–6.05)	**11.5 (5.23–21.8)**	1.34 (0.02–7.46)	0.96 (0.01–5.35)	1.19 (0.62–2.09)
All neoplasms	M	0.61 (0.41–0.87)	0.72 (0.48–1.03)	0.36 (0.17–0.65)	0.89 (0.55–1.36)	0.76 (0.63–0.90)
	F	0.49 (0.27–0.82)	0.91 (0.51–1.50)	0.81 (0.44–1.36)	0.74 (0.44–1.17)	0.64 (0.55–0.75)
Liver cancer	M	0.57 (0.06–2.04)	**3.05 (1.39–5.79)**	1.09 (0.12–3.94)	**3.53 (1.29–7.68)**	0.51 (0.18–1.10)
	F	1.66 (0.02–9.23)			4.08 (0.46–14.7)	0.38 (0.04–1.38)
Lung cancer	M	0.60 (0.19–1.39)	1.03 (0.41–2.11)		**2.26 (1.03–4.29)**	1.38 (0.98–1.88)
	F	0.34 (0.00–1.89)		0.60 (0.01–3.36)	0.40 (0.01–2.25)	0.79 (0.51–1.18)
Cervix uteri cancer	F		8.13 (0.91–29.4)	4.22 (0.06–23.50)	3.08 (0.04–17.12)	**2.67 (1.15–5.26)**
Circulatory diseases	M	0.83 (0.51–1.27)	**2.16 (1.58–2.89)**	**1.67 (1.08–2.47)**	1.06 (0.57–1.82)	0.94 (0.74–1.17)
	F	1.00 (0.32–2.32)	**3.39 (1.62–6.24)**	**2.61 (1.12–5.14)**	0.48 (0.05–1.72)	0.45 (0.28–0.70)
Ischemic heart	M	0.56 (0.20–1.22)	1.10 (0.53–2.03)	**2.53 (1.42–4.18)**	1.36 (0.54–2.80)	0.85 (0.58–1.22)
	F		1.57 (0.02–8.71)	4.33 (0.87–12.66)		0.59 (0.24–1.21)
Cerebrovascular	M	0.78 (0.16–2.29)	**4.78 (2.67–7.89)**	1.29 (0.26–3.77)	2.18 (0.59–5.57)	0.87 (0.44–1.56)
	F	1.43 (0.16–5.15)	**6.09 (1.96–14.2)**	2.31 (0.26–8.35)	1.69 (0.19–6.12)	0.49 (0.18–1.06)
External causes	M	1.04 (0.76–1.39)	0.69 (0.44–1.03)	0.74 (0.48–1.08)	0.78 (0.45–1.24)	0.94 (0.80–1.11)
	F	0.65 (0.21–1.51)	0.96 (0.31–2.24)	0.56 (0.11–1.64)	0.52 (0.10–1.52)	0.97 (0.72–1.29)
Transport accidents	M	1.29 (0.83–1.92)	0.68 (0.32–1.25)	0.75 (0.39–1.32)	0.62 (0.23–1.36)	1.06 (0.83–1.33)
	F	0.34 (0.00–1.88)	1.06 (0.12–3.81)	0.47 (0.01–2.63)	1.01 (0.11–3.66)	**1.63 (1.08–2.35)**

## Discussion

The present study demonstrates a great variation in mortality risk among immigrants in Italy by age, gender, area of provenience and specific cause of death. Overall, immigrants aged 20–59 years showed a lower mortality risk compared to Italian citizens, but this advantage (less than 15% among males and 30% among females) is smaller than what was previously found in an earlier study from another Italian region (Martini, Chellini & Sala, 2011), and similar to reports from the US and Western Europe (Singh & Hiatt, 2006; Scott & Timaeus, 2013). In particular, the increased risk of total mortality in Sub-Saharan Africans confirms data from Spain (Regidor et al., 2009) and England (Wild et al., 2007). In analyses of specific causes of death, the increased mortality for cervical cancer and the reduced mortality for breast and colorectal cancer is in line with the results of studies carried out in Sweden (Abdoli, Bottai & Moradi, 2014) and in other European countries (Arnold, Razum & Coebergh, 2010), but also with the scarce data available on cancer incidence among immigrants in Italy (Manneschi et al., 2011). For cervical cancer, screening practices (Zorzi et al., 2014) and factors affecting the access to cancer treatment may be different among immigrant women, leading to a higher mortality rate. Nonetheless, the increased mortality found among selected immigrant groups for cancer sites with low survival rates suggests that exposure to specific risk factors, rather than delayed diagnosis or worse cancer care could play a major role, such as for liver cancer (exposure to hepatitis B virus and to aflatoxin) and for lung cancer (smoking habits). The higher risk of circulatory diseases in Sub-Saharan Africans and South Asians, which has already been reported in England (Wild et al., 2007) and Spain (Regidor et al., 2009), could be explained by the high prevalence of hypertension and diabetes in these two regions (Misra et al., 2014; Soni Raleigh, 1997; Cappucio et al., 1997; Yoon et al., 2006). Therefore, it becomes very important from a public health perspective to monitor such specific risk profiles, e.g., for better surveillance and control of blood pressure. This can help policy-makers to develop priorities for primary and secondary prevention. For example, for circulatory disorders, physical activity and a healthy diet should be promoted, and known risk factors (hypertension, diabetes) should be better controlled at a primary care level; for cancer, participation in cervical cancer screening programs should be increased, and anti-smoking campaigns should be reinforced and tailored towards ethnic groups at a higher risk.

The mortality ratio between the immigrant and the Italian population greatly varies with age. We found an increased mortality among immigrant children, especially in the youngest age class (0–4 years). An increased infant mortality rate among immigrants is a well-known phenomenon in Italy (Martini, Chellini & Sala, 2011). Previous studies have shown that infant mortality rates depend on the immigrant’s destination country, and that different effects are associated with different source countries (Naimy et al., 2013). In Italy, as in other Western countries, an increasing proportion of births are from immigrant women coming from world regions where low birth weight and infant death are more frequent (Sobotka, 2008).

The risk of death remains higher through all pediatric age classes (females) and also in young adults (males). Thereafter, the mortality gap favorable to immigrants becomes larger with age. This pattern has already been reported in the Netherlands (Bos et al., 2007), with a similar definition of the immigrant population (including second-generation immigrants). Moreover, the underestimation of mortality rates among immigrants has been reported to be more probable in older age classes, mainly due to unregistered remigration to the country of origin (Spallek et al., 2012). Within this scenario, all methods of age-standardization are inadequate, leading to different rate ratios when applying different standards. Therefore, we chose to truncate estimates at 59 years of age. We decided also to exclude from the analysis the pediatric population based on two main reasons: causes of death (mainly perinatal and congenital causes in ICD-10) are usually specific and non-comparable with the adult population, and available data on mortality from cancer and circulatory diseases among immigrants in Europe are generally restricted to the adult population. Furthermore, through indirect standardization, weights were chosen from the study (immigrant) populations, and SMR estimates allowed to explore cause-specific mortality rate ratios across different immigrant groups also when dealing with small numbers.

As the census takes place only every 10 years, restricting the analysis of mortality to years close to the census helped to deal with reliable denominators for mortality rates (Wild et al., 2007). The use of census data could have introduced a numerator/denominator bias. However, this bias should be of limited size in analyses by large area of provenience and not by single country of citizenship (Regidor et al., 2008). In particular, the use of census data allows to limit the magnitude of some biases that usually lead to an underestimation of mortality in immigrants, namely the mobility bias (immigrants spend shorter or longer periods in the country of origin), and unregistered remigration (delays in registration in municipal registries of the final return to the country of origin), which usually inflate the mortality rate denominators. In particular, the remigration can be differential, affecting especially immigrants with health problems and/or low socio-economic profiles (unhealthy remigration effect). Furthermore, during periods of global economic crisis, both remigration to the country of origin and a new emigration towards other countries with more job opportunities may increase.

We excluded from our analyses immigrants who are not residents but are legally present in the study area, as well as illegal immigrants. These subjects are estimated to account for another 10–15% of the immigrant population in Italy (Blangiardo, 2013). Due to the lack of estimates by age, gender or area of provenience, the study was restricted to legal residents.

One limitation of the study is the absence of data on socioeconomic status and on time since immigration. Some studies found death rates to be substantially influenced by socio-economic status in immigrants (Bos et al., 2004; Rostila & Fritzell, 2014; Abdoli, Bottai & Moradi, 2014), and socio-economic inequalities are considered a sizeable cause of inequalities in mortality by country of origin.

The length of time of residence in the host country is assumed to be a proxy for acculturation; at the same time, it could be expected that with time spent in the host country, immigrant groups increasingly assume specific risk patterns of the native population. Such changes are difficult to predict in the presence of rapidly changing dynamic populations (Bos et al., 2004): new subjects undergoing selective immigration patterns continue to contribute to the growth of immigrant groups; moreover, the global process of westernization of dietary habits and other risk factors is heavily involving most emigration countries, and many young adults coming to Europe have probably already acquired risk profiles associated with nutritional habits and metabolic attributes in their country of origin (Misra & Ganda, 2007). To address the transition of mortality patterns, it would be necessary to compare mortality figures also with those of the countries of origin; however only few previous studies were able to collect relevant data to investigate this issue (Spallek et al., 2012). Furthermore, it should be taken into account that the health status of different populations of immigrants could depend on differences not only in the exposure to risk factors in their country of origin, but also in reasons for migration and migration pathways due to geographical distance or other barriers.

Within this context, surveillance of mortality by cause is of paramount importance to set priorities for both research and public health. The present data reduce the informative gap on mortality among immigrants in Italy, highlighting the need for further analyses at both the regional and national levels.

## Conclusion

Our results have shown important variations in mortality by area of provenience, in most cases similar to what has been reported in studies performed in traditional immigration countries of North America and other parts of Europe. Specifically, mortality from circulatory diseases emerges as a public health concern, especially in selected immigrant groups. These data support a recent multidisciplinary call for more detailed data on cardiovascular risk factors aimed at a better control of circulatory diseases among immigrants in Italy (Modesti et al., 2014). A continual monitoring of immigrants’ health is crucial since its improvement will have a substantial impact on the population’s overall health and on the magnitude of health inequalities (Singh & Hiatt, 2006).
